# Preclinical prototype validation and characterization of a thermobrachytherapy system for interstitial hyperthermia and high-dose-rate brachytherapy

**DOI:** 10.1016/j.phro.2024.100606

**Published:** 2024-06-27

**Authors:** Ioannis Androulakis, Rob M.C. Mestrom, Sergio Curto, Inger-Karine K. Kolkman-Deurloo, Gerard C. van Rhoon

**Affiliations:** a Department of Radiotherapy, Erasmus MC Cancer Institute, University Medical Center, Rotterdam, the Netherlands; b Department of Electrical Engineering, Eindhoven University of Technology, Eindhoven, the Netherlands

**Keywords:** Brachytherapy, Electromagnetic heating, Hyperthermia, Radiation therapy, Validation

## Abstract

**Background and purpose:**

Integrating simultaneous interstitial hyperthermia in high-dose-rate brachytherapy treatments (HDR-BT) is expected to lead to enhanced therapeutic effect. However, there is currently no device available for such an integration. In this study, we presented and validated the thermobrachytherapy (TBT) preclinical prototype system that is able to seamlessly integrate into the HDR-BT workflow.

**Materials and methods:**

The TBT system consisted of an advanced radiofrequency power delivery and control system, dual-function interstitial applicators, and integrated connection and impedance matching system. The efficiency and minimum heating ability of the system was calculated performing calorimetric experiments. The effective-heating-length and heating pattern was evaluated using single-applicator split phantom experiments. The heating independence between applicators, the ability of the system to adaptable and predictable temperature steering was evaluated using multi-applicator split phantom experiments.

**Results:**

The system satisfied interstitial hyperthermia requirements. It demonstrated 50 % efficiency and ability to reach 6 °C temperature increase in 6 min. Effective-heating-length of the applicator was 43.7 mm, following the initial design. Heating pattern interference between applicators was lower than recommended. The system showed its ability to generate diverse heating patterns by adjusting the phase and amplitude settings of each electrode, aligning well with simulations (minimum agreement of 88 %).

**Conclusions:**

The TBT preclinical prototype system complied with IHT requirements, and agreed well with design criteria and simulations, hence performing as expected. The preclinical prototype TBT system can now be scaled to an in-vivo validation prototype, including an adaptable impedance matching solution, appropriate number of channels, and ensuring biocompatibility and regulatory compliance.

## 1 Introduction

High-dose-rate brachytherapy (HDR-BT) is a well-established method for highly localized and hence highly hypofractionated radiotherapy treatments [1]. Such treatments can take the form of a localized boost in conjunction with External Beam Radiotherapy (EBRT) [2], [3], or a standalone highly hypofractionated monotherapy [4]. However, there are limits to the extent of hypofractionation that can be effectively employed [5]. These limitations arise from both biology and physics [6].

Hence, there is a need to explore alternative strategies for improving cell kill rates, one of which is interstitial hyperthermia (IHT). IHT is currently utilized sequentially after HDR-BT for the treatment of prostate cancer [7], [8] and breast cancer [9]. The efficiency of this combined treatment could be further enhanced if they were applied concurrently, as research indicates that the greatest thermal enhancement is achieved when hyperthermia is administered concurrently with radiation therapy [10]. The development of a system capable of facilitating such simultaneous treatment offers interesting prospects in terms of clinical efficiency and patient comfort, such as potentially reducing the overall number of treatment sessions and treatment duration, while realizing equal tumor outcome and low toxicity.

There have been attempts in the past to develop applicators that would facilitate simultaneous microwave and ultrasound based IHT and HDR-BT [11], [12]. In the former case, temperature distribution cannot be controlled longitudinally, while the latter case, it has proven difficult to sufficiently miniaturize the applicators to what is currently an acceptable catheter size in interstitial HDR-BT. Therefore, in our previous research, we designed the thermobrachytherapy (TBT) dual function applicators as a means to incorporate simultaneous capacitive coupling IHT (CC-IHT) into HDR-BT treatments [13]. We also developed a rapid simulation method for these applicators for efficient treatment planning [14], and an approach to optimize simultaneous TBT treatment plans [15]. This approach could lead to a significant dose reduction in the organs at risk by achieving the same effective radiation dose to the target using only 80 % of the original physical HDR-BT dose.

The previous CC-IHT system, had known limitations like low impedance independence between channels, and no possibility to change the phase setting of each channel during treatment, leading to inefficient and suboptimal treatments [16], [17]. To bring the TBT applicators to clinical practice [13], [14], [15], a treatment delivery system that can overcome those issues needs to be designed. In addition, the system must comply with the latest European Society of Hyperthermic Oncology IHT quality assurance guidelines (ESHO-IHT-QA) [18].

In the current study, we developed a preclinical prototype TBT system. The final system consisted of three main components: a radiofrequency power delivery and control system, an integrated connection and impedance matching system, and dual-function interstitial applicators. The feasibility, characterization and validation of the system to operate according to ESHO-IHT-QA was demonstrated in an ex vivo setting, i.e. technology readiness level 4 (TRL4) [19], from which it followed that the system was able to execute adaptable and predictable heating patterns.

## 2 Materials and methods

### 2.1 System description

**Fig. 1:**
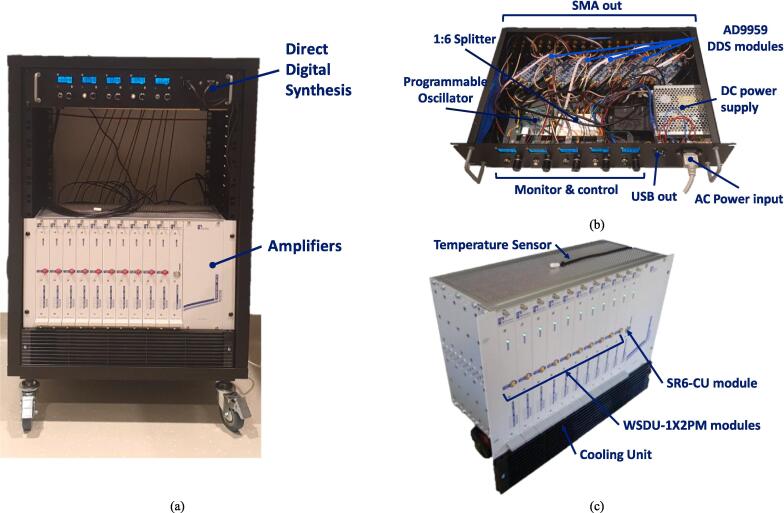
Overview of the power delivery and control system; (a) the complete system mounted on a mobile 16U rack; (b) photograph of the direct digital synthesis modules; (c) photograph of the amplifier modules. For a more detailed description of the system, refer to the Supplementary Materials.

An overview of the radiofrequency power delivery and control system can be seen in Fig. 1. The system consisted of two main blocks; the custom signal generation system which generated the 27 MHz signal using direct digital synthesis (DDS), and the power amplification system (Becker Nachrichtentechnik, Germany), which consisted of variable high-power amplifiers, which can amplify the signal up to 5 W output power per single channel. The system has integrated forward (FWD) and reflected (REFL) power sensors (Minicircuits, USA), in order to monitor the actual power output of each of the 10 channels. All components were connected to and continuously controlled through a desktop computer. A more detailed overview can be found in the Supplementary Materials.

The design of the dual-function interstitial applicator was based on previously published research [13]. With the electrode constructed around the standard flexible clinical HDR-BT catheter, the applicator maintained a fully unoccupied inner lumen for the HDR-BT source. On the outer wall of the catheter, two 20 mm long copper electrodes were positioned, maintaining 5 mm separation between them. For proper insulation the electrodes were coated with a thin polytetrafluoroethylene (PTFE) dielectric layer. The electrodes were individually linked by copper lines to connection patches located at the catheter's proximal end. These connection patches facilitated connection to the power delivery system.

**Fig. 2:**
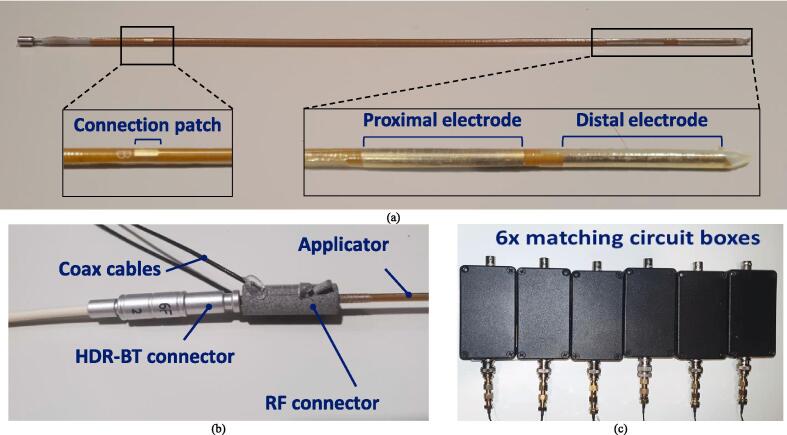
Overview of (a) the TBT Applicator; (b) the Connectors on the applicator; (c) the matching network boxes.

For the fabrication of the dual function interstitial applicators (Fig. 2.a), copper electrodes (18 μm thickness) and connection lines (18 μm thickness and 0.2 mm width) were printed on flexible polyimide sheets (80 μm thickness) using lithography and covered with an immersion gold finishing (PCBWay.com Ltd, PRC). The flexible circuit was rolled on a 6 Fr (2 mm outer diameter) polyoxymethylene (POM) needle-shaped brachytherapy catheter (Elekta AB, Stockholm, Sweden). For assembly stabilization, as well as to prevent galvanic contact between human tissue and conductive components, a thin PTFE tube with an inner diameter of 2.1 mm and a typical wall thickness of 19 μm (Zeus Industrial Products, USA) was slid over the flexible circuit. Additionally, the PTFE tube was closed on the sharp edge of the brachytherapy catheter (using an overhand knot) to insulate the electrode from the target tissue or material.

To establish electrical connections between the power delivery system and the two connection patches of each applicator, a custom connector was developed. For each applicator, a pair of 0.5 m long 50 Ω coaxial cables with an outer diameter of 1.1 mm (W9020M, Pulse Electronics) were connected on one end to SMA connectors, and on the other end to a custom Polyamide 12 connector created through multi-jet fusion 3D printing (Materialise NV, Belgium). The connector was designed with a click-system, enabling a seamless attachment of power lines to the applicator connection patches, while maintaining the proximal end of the applicator free for connection to the HDR-BT afterloader (Fig. 2.b). The ability of the connectors to connect to the applicators without compressing the inner diameter of the applicator lumen was tested by passing a dummy brachytherapy source cable (Flexitron Afterloader, Elekta AB, Stockholm, Sweden) when the connectors were attached to the applicator.

Impedance matching networks were placed between the custom connector cables and the power system. The individual impedance matching networks were L-type with a shunt tunable capacitor (9–200 pF) followed by a series inductor (∼1 µH) and a series tunable capacitor (9–200 pF). The impedance matching networks were encapsulated in electromagnetically shielded boxes and individually tuned for each experiment (Fig. 2.c). The individual impedance matching networks could be tuned to a passive input reflection coefficient of <−20 dB. When connected to the power system, a lower active return loss could be observed. The active input reflection coefficient could be maintained <−6 dB in all evaluated cases.

### 2.2 Validation and characterization methodology

**Fig. 3:**
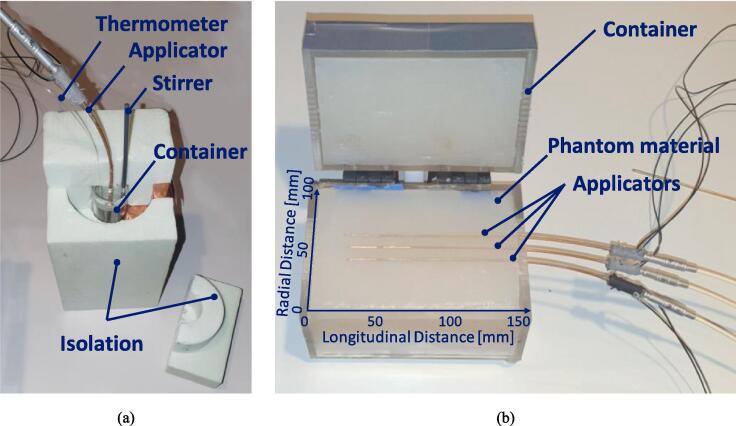
Overview of experimental setups. (a) Calorimetric experiments (b) split phantom with three applicators inserted at an in-between distance of 10 mm.

Calorimetric experiments were performed to evaluate the effective power delivery and applicator efficiency, as described in ESHO-IHT-QA [18]. The applicator was inserted into a cylindrical container with a 0.4 % saline solution. The container had a 25 mm inner diameter, 96.5 mm height (volume $V=47.4cm^{3}$), and three openings on top for hyperthermia applicator, temperature measurement probe, and stirrer insertion. The entire setup was enclosed within a Styrofoam isolation box to minimize thermal losses, as shown in Fig. 3.

To characterize the individual applicator's performance, the applicator was activated for a duration of 6 min with stable input power. Temperature measurements started 5 min before heating initiation and extended for 5 min after heating completion. The temperature was measured using a single point fiber-optic temperature probe and FT1310 thermometry readout system (Takaoka Electric, Tokyo, Japan). Throughout the temperature acquisition process, the saline solution was continuously agitated using the incorporated stirrer, in order to ensure temperature homogeneity of the saline solution. The experiment was repeated 3 times using different power levels. Following ESHO-IHT-QA, the system needs to demonstrate its ability to heat 6 °C within 6 min of heating.

Efficiency (η) was defined as effective power ($P_{\mathrm{eff}}$) over input power ($P_{\mathrm{in}}$), and was calculated from the relation

(1) $${\eta =P}_{\mathrm{eff}}/P_{\mathrm{in}}=\frac{\rho \;c_{w}V}{P_{\mathrm{in}}}\frac{\Delta T}{\Delta t}$$

where ρ was the density of the solution, $c_{w}$ the specific heat capacity of the solution, V the volume, ΔT the temperature increase achieved during heating the continuously agitated saline solution, and Δt the heating time [18]. For the 0.4 % saline solution $\rho =1000\;kg/m^{3}$ and $c_{w}=4158\;J/kg\;K$ was used [20].

We conducted heating experiments using a 150 × 100 × 100 mm^3^ rectangular phantom container filled with a solid agar phantom mixture (1 L of deionized water, 4 g of NaCl, and 40 g of agar) that resembles the electromagnetic properties of water-based tissue (electrical conductivity of 0.84 S/m and relative electrical permittivity of 74.4 at room temperature of 20 °C). The phantom was designed to be divided into two separate sections, forming a so-called split phantom. In this setup, the applicators were completely embedded within the lower section of the split phantom. This ensured that all applicators were completely enveloped by the phantom material, ensuring optimal contact.

We explored three distinct setups: In the first setup, we used a single applicator to test its individual behavior. In the second setup, an array of three applicators was positioned in parallel, spaced at an intermediate distance of 20 mm as suggested by ESHO-IHT-QA [18] for heating pattern independence testing. The third setup involved placing the three applicators closer to each other, maintaining the clinically recommended spacing of 10 mm between them.

Across all three setups, we placed a single-point fiber-optic temperature probe at a depth of 5 mm beneath the central point of each applicator's heating length. This placement allowed for temporal temperature monitoring throughout the experiments. For spatial temperature monitoring, after turning off the power, the top phantom part was removed and the temperature distribution was imaged using a T1020 infrared camera (FLIR, Wilsonville, ON, USA) with a thermal sensitivity of <20 mK and accuracy of 1 °C. The spatial resolution of the IR measurements was kept between 0.38 and 0.42 mm.

In the first experimental setup the applicator was heated for 3 min with 3 W applied to both electrodes. The effective heating length of each of the two electrodes and the two electrodes combined was measured. The effective heating length was defined as the length at which the temperature rise is 50 % or more of the maximum temperature rise measured at a distance of 5 mm from the applicator center [18]. Furthermore, the heating pattern was defined as the $T_{\max}/e$ *iso*-temperature contour where $T_{\max}$ is the maximum temperature [21].

In the second experimental setup the overlap of the $T_{\max}/e$ *iso*-temperature contours created by one applicator in three different scenarios was calculated. In the three scenarios power was applied to only one lateral applicator (1 0 0), two lateral applicators (1 0 1), all three applicators (1 1 1) [18]. According to ESHO-IHT-QA, the variation between the three scenarios should be bellow ±20 %.

The third experimental setup was used to investigate the system’s ability to create different heating patterns using different phase and amplitude settings (Table 1), and to investigate whether the heating patterns agree with predictions. The latter was done by calculating the overlap between experimental and simulated $T_{\max}/e$ *iso*-temperature contours.

**Table 1:** Phase and amplitude settings of performed temperature steering experiments and corresponding simulations.

Applicator	Top				Middle				Bottom			
Electrode	Distal		Proximal		Distal		Proximal		Distal		Proximal	
Settings	Power (W)	Phase (°)	Power (W)	Phase (°)	Power (W)	Phase (°)	Power (W)	Phase (°)	Power (W)	Phase (°)	Power (W)	Phase (°)
Proximal	0	−	3	180	0	−	3	0	0	−	3	180
Distal	3	0	0	−	3	180	0	−	3	0	0	−
Distal-Proximal	3	0	3	180	3	180	3	0	3	0	3	180
Middle	3	0	3	180	3	0	3	180	3	0	3	180
Diagonal	3	0	0	−	3	180	3	0	0	−	3	180
Star	3	0	0	−	3	0	3	180	0	−	3	180

Full wave electromagnetic and transient thermal simulations were performed using the finite-difference time-domain (FDTD) solvers of Sim4Life (Zurich MedTech AG, Switzerland) as described in previously published research [13]. The simulations only included the applicators, without taking impedance matching and cabling into consideration. In the comparison with the experimental results, the temperature simulation results were scaled so that the 99th percentile of the temperature in the simulation matches the measured temperature.

## 3 Results

### 3.1 Efficiency

**Fig. 4:**
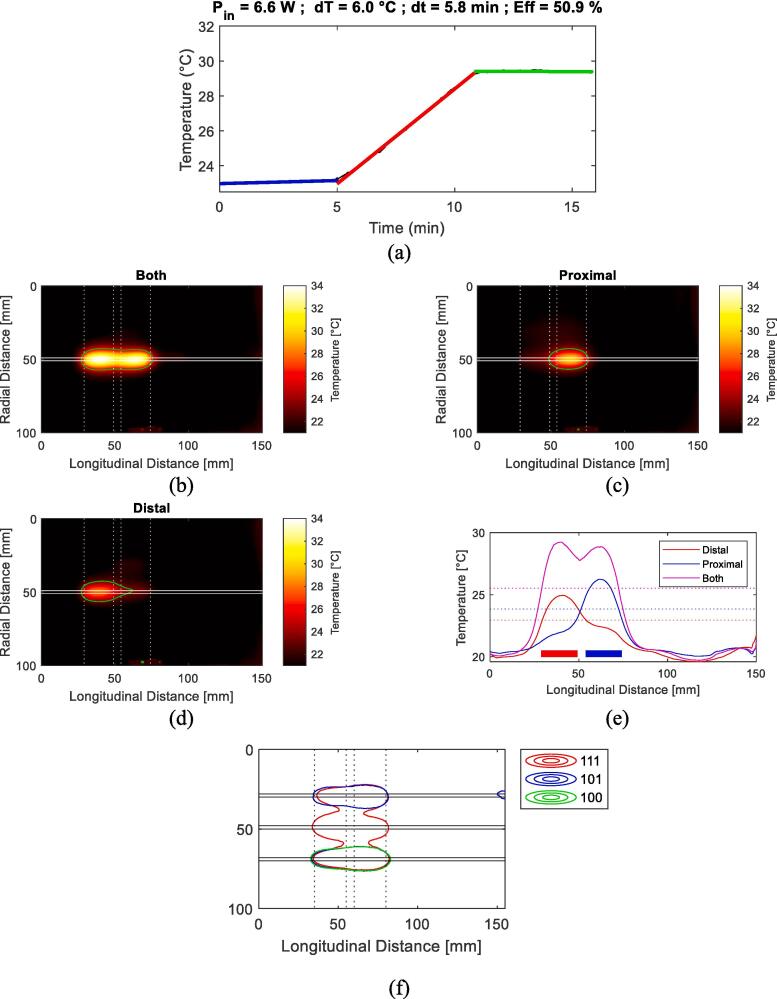
(a) Shows temperature measurements before, during, and after power application in a calorimetric experiment. (b–d) Display IR images of single applicator experiments with 3 W applied for 3 min on (b) both, (c) only proximal, (d) only distal electrodes. Horizontal lines mark applicator edges, vertical dotted lines mark electrode edges. (e) Illustrates temperature along an axis parallel to the applicator in experiments b-d. Horizontal dotted lines indicate effective heating length. (f) Presents heating pattern independence experiments with $T_{\max}/e$*iso*-temperature contours observed in IR images after 3 min of heating with 3 W applied on both electrodes of one lateral applicator (1 0 0), two lateral applicators (1 0 1), all three applicators (1 1 1). Horizontal lines mark the applicator edges, vertical dotted lines mark electrode edges.

The three calorimetric experiments with a constant total power of 6.1 W, 6.6 W, 8.7 W resulted in an efficiency (η) of 50.0 %, (48.7 %–50.9 %). The applicator fulfilled the minimum requirement to reach a 6 °C temperature increase in 6 min, as can be seen in Fig. 4.a.

### 3.2 Effective heating length and heating pattern

An effective heating length of 43.7 mm when both electrodes were turned on. The effective heating length of the single electrodes was 24.7 mm and 21.7 mm for the distal and proximal electrode respectively. The heating pattern, expressed as the $T_{\max}/e$ *iso*-temperature contours showed that the two electrodes created similar heating patterns, and that the heating pattern did not change when power was simultaneously applied on both electrodes. The heating pattern is visualized in Fig. 4.b–d, where it is visible that heating extends to the applicator tip.

### 3.3 Heating pattern independence

In the experimental setup with three applicators at a 20 mm spacing, the three resulting heating patterns, defined by the $T_{\max}/e$ *iso*-temperature contours, exhibited stability. This can be observed in Fig. 4.f. Specifically, we observed a 97 % overlap between the heating pattern of the first applicator in cases ‘100–101’ and an 89 % overlap between cases ‘100–111’. This overlap was well within the ±20 % acceptable variation.

### 3.4 Temperature steering

**Fig. 5:**
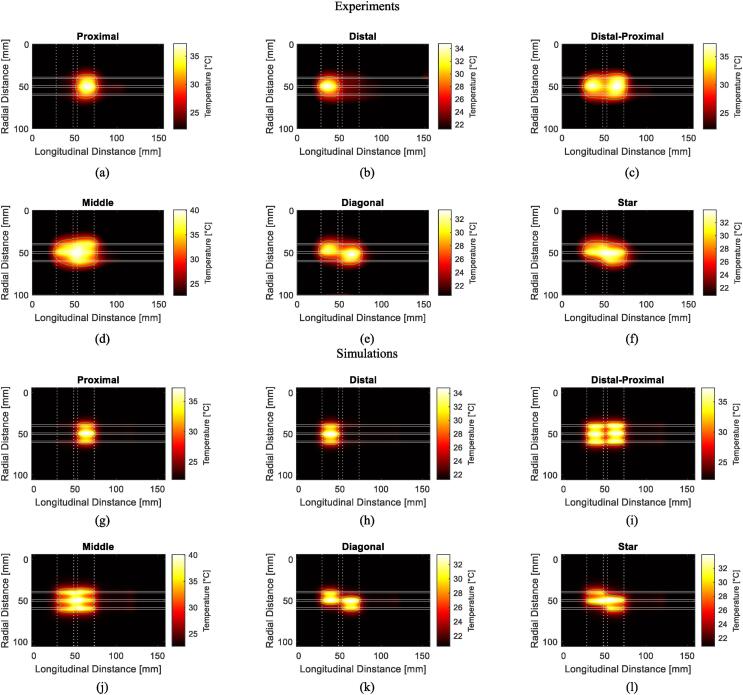
Temperature steering experiments. (a–f) IR images after 3 min of heating with phase and amplitude settings presented in Table 1. (g–l) Simulation results of the same plane after 3 min of heating with phase and amplitude settings presented in Table 1. Each of the six subplots (a–f) and (g–l) corresponds to one row of settings in Table 1. The horizontal continuous lines correspond to the edges of the applicators. The vertical dotted lines correspond to the edges of each electrode.

In the experimental setup with three applicators at a clinically relevant 10 mm spacing, the phase and amplitude settings in Table 1 resulted in the temperature distributions in Fig. 5. The system showed capable of transitioning between partial proximal (Fig. 5.a) or distal (Fig. 5.b) electrode heating as well as activating the entire effective heating length (Fig. 5.c and d). Within this context, adjustments to the phase settings resulted in distinct heat distributions. Furthermore, the system achieved alternative heating patterns, including diagonal heating and star-shaped heating patterns (Fig. 5.e and f). Comparing the experimental results of Fig. 5.a–f with the simulations of Fig. 5.g–l, an $T_{\max}/e$ *iso*-temperature contour overlaps of 94 %, 89 %, 93 %, 91 %, 88 %, 89 % were observed for the proximal, distal, distal-proximal, middle, diagonal, star heating, respectively.

## 4 Discussion

In this study we presented, validated and characterized the TBT system prototype, which consisted of an RF power delivery system, dual function interstitial applicators, a tunable matching network and a custom RF connection system that allowed for simultaneous connection of the applicators to the RF system and to the HDR-BT afterloader. We characterized the system in terms of efficiency, effective heating length, and showed that it satisfied the minimum requirements for a clinical IHT device as defined in ESHO-IHT-QA [18]. Finally, we showed that the applicators could produce different heating patterns when the phase and amplitude settings of each electrode were altered, and that these heating patterns agreed well with simulations.

The hardware configuration we developed for this study was highly flexible, enabling precise control over power and phase steering. This flexibility is a significant advantage as it allows the system to be tailored to specific clinical needs that may emerge from future research. Furthermore, the ability to independently control power and phase steering for each electrode avoids the issues encountered with the previous CC-IHT system (MECS) [16]. In this previous system, power was controlled per groups of four electrodes, phase was stable per electrode, and impedance imbalance was a common issue due to the low isolation between channels within the same group [16]. This impedance imbalance easily led to an amplitude imbalance, resulting in unpredictable heating patterns. Our new configuration addresses these challenges, leading to more reliable and predictable results. The incorporation of forward and reflected power measurement within the system introduces the prospect of an automated active impedance matching system. This future development will have the potential to significantly enhance the impedance matching capabilities of the system beyond the matching achieved in the current configuration (up to 6 dB return loss). The automation of the impedance matching process also represents a promising way to further simplify the treatment delivery process.

A key feature of this system was the seamless integration of the TBT system within a brachytherapy procedure using a custom connector. This allows for simultaneous use of the two modalities. The system also included a reproducible and simple applicator fabrication technique, which could lead to single-use applicators. While manual fabrication has been demonstrated, further refinement, in vivo testing, and considerations for sterilization and commercial mass production will require future investigation.

In terms of system performance, we found that the TBT dual function applicators satisfied the requirements defined in ESHO-IHT-QA [18]. The applicators exhibited heating efficiency (50 %), which was within the range of current commercial microwave applicators for sequential application of brachytherapy and hyperthermia [22]. However, there is still room for improvement, especially if automatic active impedance matching is implemented, which would minimize mismatch losses. Future versions of the system should include this proposed impedance matching solution, which will improve heating efficiency and likely further improve agreement with simulations. The effective heating length (43.7 mm) of the applicators aligned well with the designed specifications (45 mm) in our earlier study [13]. Notably, this EHL was in close alignment with the existing brachytherapy procedure, ensuring that the entire area being irradiated can be heated, extending up to the distal tip of the applicator. This heating length was chosen to meet the clinical needs for whole prostate heating, but could also be adapted for heating of other targets. Moreover, our experiments have shown that the applicators were capable of maintaining sufficient decoupling when placed at a spacing of 20 mm (min $T_{\max}/e$ *iso*-temperature contour overlap: 89 %).

At the clinically relevant applicator spacing of 10 mm, the system exhibited considerable flexibility in heating patterns. More importantly, these heating patterns agree with simulated heating patterns (minimum $T_{\max}/e$ *iso*-temperature contour overlap: 88 %). This flexibility holds promise for tailoring treatments to complex target shapes and for optimizing the thermal distribution within the target area.

In conclusion, we presented an advanced (TRL4) preclinical prototype TBT system, which has the ability to provide highly adaptable CC-IHT simultaneously with HDR-BT. The prototype system was tested ex-vivo, passing the requirements for IHT systems, and the experimental characterization showed the system behaved as initially designed. The preclinical prototype TBT system is now ready to be scaled to an in-vivo validation prototype. This next phase will involve improving heating efficiency with an adaptable impedance matching solution, increasing the number of channels for clinical relevance, ensuring biocompatibility and regulatory compliance, and importantly, designing RF electronics that are robust to radiation exposure.

## Funding

This research was funded by Elekta AB, Stockholm, Sweden (grant number 106932, task 4). The funders had no role in the study design, the collection, analysis or interpretation of data, the writing of the report and the decision to submit the article for publication.

## CRediT authorship contribution statement

**Ioannis Androulakis:** Conceptualization, Methodology, Software, Validation, Formal analysis, Investigation, Data curation, Writing – original draft, Visualization, Project administration. **Rob M.C. Mestrom:** Conceptualization, Methodology, Writing – review & editing, Supervision. **Sergio Curto:** Conceptualization, Methodology, Resources, Writing – review & editing, Supervision, Funding acquisition. **Inger-Karine K. Kolkman-Deurloo:** Conceptualization, Methodology, Resources, Writing – review & editing, Supervision, Funding acquisition. **Gerard C. van Rhoon:** Conceptualization, Methodology, Resources, Writing – review & editing, Supervision, Project administration, Funding acquisition.

## Declaration of competing interest

The authors declare the following financial interests/personal relationships which may be considered as potential competing interests: I. Androulakis, R.M.C. Mestrom, I.K.K. Kolkman-Deurloo, and G.C. van Rhoon are inventors of a pending patent on an interstitial hyperthermia device (WO2022235155A1). S. Curto declares no conflict of interest.
