# Supplementary material for: Preclinical prototype validation and characterization of a thermobrachytherapy system for interstitial hyperthermia and high-dose-rate brachytherapy
Source: Phys Imaging Radiat Oncol. 2024 Jun 27;31:100606. doi: 10.1016/j.phro.2024.100606 (PMC11294723; doi:10.1016/j.phro.2024.100606)
Supplement: Supplementary Data 1 [file mmc1.pdf]

SUPPLEMENTARY MATERIALS
to
Thermobrachytherapy system for simultaneous interstitial hyperthermia and high-dose-rate brachytherapy: Preclinical prototype validation and characterization

Ioannis Androulakis*[1], Rob M.C. Mestrom[2], Sergio Curto[1], Inger-Karine K. Kolkman-Deurloo[1], Gerard C. van Rhoon[1]

[1] Department of Radiotherapy, Erasmus MC Cancer Institute, University Medical Center, Rotterdam, The Netherlands
[2] Department of Electrical Engineering, Eindhoven University of Technology, Eindhoven, The Netherlands
*Corresponding Author: i.androulakis@erasmusmc.nl

Radiofrequency Power Delivery and Control system description

The custom built power delivery and control system architecture can be seen in diagram of Fig. A.1. A description of individual components is described in the following subsections.

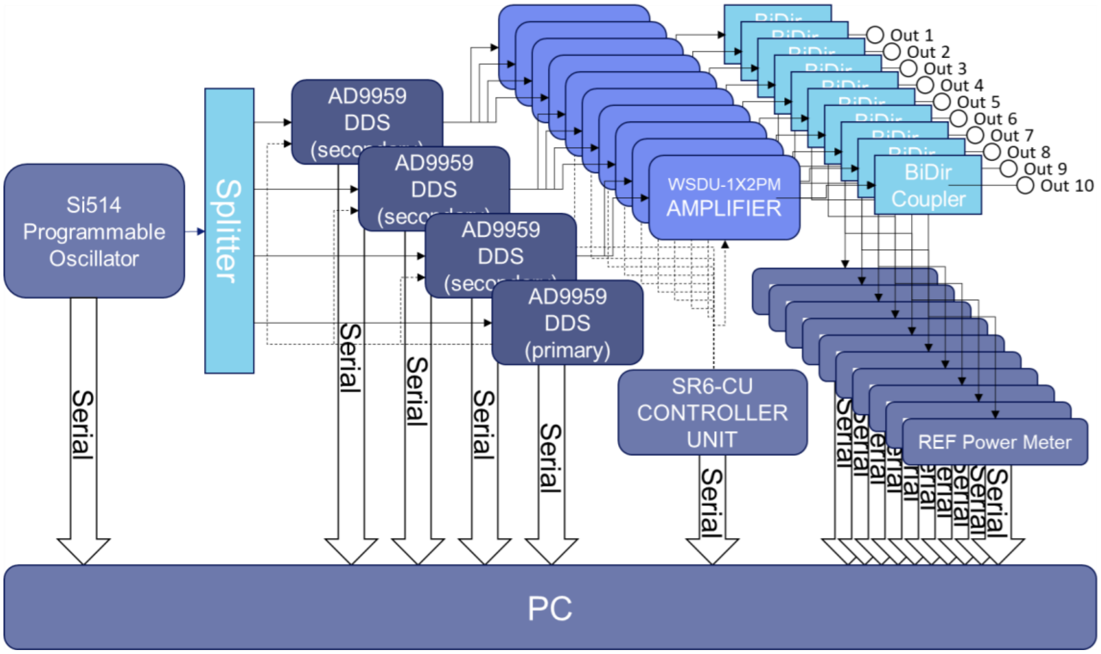

Fig. A.1. diagram of the power delivery and control system.

Direct Digital Synthesis

The DDS system is based on five AD9959 multichannel frequency synthesizer integrated circuits (Analog Devices, USA), each mounted on its own circuit board and controlled through an ATMega2560 microcontroller (Fig. 1.c). Each AD9959 integrated circuit can produce four digitally synthesized coherent output signals, with independent frequency, phase, and amplitude modulation. To synchronize all five synthesizers, an external 125 MHz reference signal, generated by an Si514 programmable oscillator (Skyworks Solutions, USA), is given as input to the five integrated circuits, and the system clocks of four synthesizers (secondary) are programmatically synchronized to the fifth one (primary). The latter is done by an automatic synchronization method integrated in the AD9959 synthesizer, that which sends a synchronization pulse from the controller synthesizer (SYNC OUT) to the worker synthesizers (SYNC IN).

The DDS system can produce 10 synchronous signals with a frequency of 27 MHz and a phase shift in the range of 0-360° with a step size of 0.1°. The signal amplitude can be varied between -60 dBm and -7 dBm in steps of 1 dBm. The settings can be monitored and controlled manually or with a PC using a serial communication port.

High Power Amplifiers

The high-power amplification system consists of ten WSDU-1x2PM amplifiers (Becker Nachrichtentechnik, Germany)

controlled by a SR6-CU controller unit (Becker Nachrichtentechnik, Germany) (Fig. 1.c). The amplifier modules consist of a 2-way multicoupler with a variable output power of up to 5 W per channel. The modules have a typical variable gain ranging from +19.25 dB to +51 dB at the frequency of 27 MHz, and a resolution of 0.25 dB. Furthermore, the modules have integrated forward power sensors with a typical accuracy of 0.3 dB. The controller unit communicates with a PC using serial communication.

Power detection and control

Whilst forward power sensors are integrated in the amplifier modules, reflected power sensors are not. Therefore, an external power sensor is connected at the output of each amplifier, using a ZFBDC20-62HP-S+ 20 dB Bi-directional coupler (Minicircuits, USA). The PWR-4GHS power sensor (Minicircuits, USA) can measure power on a wide range of power levels (1 μW to 0.1 W), covering the whole range of possible power outputs and being able to measure reflected power levels down to -10 dBm (given the -20 dB coupling). This gives the system the ability to evaluate the reflection coefficient even at low power outputs with a maximum power measurement uncertainty of ±0.35 dB.

For proper power leveling, a calibration of the power system is performed with all outputs connected to 50 Ω loads. With a polynomial fit in the power range of operation, the desired forward power level can be matched instantly given that the input frequency and amplitude remain stable. When a mismatch occurs, the power is adapted with a proportional–integral–derivative controller (PID controller) to the desired effective output power.
